# Examination of the transport characteristics of pediatric trauma patients

**DOI:** 10.55730/1300-0144.5856

**Published:** 2024-06-11

**Authors:** Ramiz YAZICI, Muhammed GÜNER, Efe Demir BALA, Ayşe Fethiye BASA KALAFAT, Eyüp SARI, Salih FETTAHOĞLU, Rabia Birsen TAPKAN, Utku Murat KALAFAT, Aziz Ahmet SÜREL, Serkan DOĞAN

**Affiliations:** 1 Department of Emergency Medicine, İstanbul Kanuni Sultan Süleyman Training and Research Hospital, University of Health Sciences, İstanbul, Turkiye; 2 Department of Pediatrics, Gülhane Faculty of Medicine, University of Health Sciences, Ankara, Turkiye; 3 Department of General Surgery, Ankara Bilkent City Hospital, University of Health Sciences, Ankara, Turkiye

**Keywords:** Pediatric trauma, secondary triage, prehospital, transport, injury

## Abstract

**Background/aim:**

Injury is an important public health problem in the pediatric age group and one of the leading global causes of morbidity and mortality. The fact that pediatric trauma has a significant impact on patients, families, and countries shows the need for a better understanding of this phenomenon. This study investigates the demographic characteristics, reasons for admission to the hospital, and diagnoses of pediatric trauma patients who received prehospital emergency health services.

**Materials and methods:**

This study was designed as a retrospective observational study and included all patients under the age of 18 who received emergency healthcare due to trauma and were registered in the Emergency Health Automation System after a call was placed to the emergency call center between 1 January 2018 and 31 December 2022. Information such as the reason for calling an ambulance, ICD-10 diagnosis codes, mechanism of injury, time of arrival at the scene, transport duration from the scene to the hospital, and reasons for interfacility transfers were collected for all patients.

**Results:**

A total of 37,420 patients were included in the analysis. Seventeen patients were found dead at the scene of the trauma and 35 patients experienced cardiac arrest on the way to the hospital from the scene. The difference between age groups in terms of time from arrival at the scene to arrival at the hospital was statistically significant (p < 0.001). Falls were the most common cause of trauma in all age groups, followed by traffic accidents. Patients requiring a specialist and transferred primarily for fall-related injuries were in direct proportion to the total number of cases (65.0%, n = 1838), followed by cases of traffic accidents and sports injuries. Most of the secondary transports were made to a training and research hospital or state hospital.

**Conclusion:**

Targeted preventive measures and community education should address the specific causes of trauma that are more prevalent in certain age groups. Early identification of special patient groups that typically require secondary transport can reduce mortality and morbidity related to trauma by facilitating direct transfers to appropriate hospitals.

## 1. Introduction

Injury is a major public health issue in the pediatric age group and is one of the leading causes of morbidity and mortality worldwide [1,2]. A study conducted in 2013 estimated that of the more than 900 million patients who sustained injuries, 4.8 million died [3]. According to a report published by the Turkish Statistical Institute (TÜİK) in 2023, in Türkiye, the most common causes of child deaths were injury and poisoning in 2020 and 2021. According to the TÜİK data, 5882 and 6122 child deaths were reported in the pediatric age group in 2020 and 2021, respectively. Of these children, 22.9% in 2021 and 21.4% in 2022 died due to injury or poisoning.^1^

Pediatric trauma is a phenomenon drawing significant attention not only because it leads to death but also because it causes lifelong disabilities in many survivors. The rate of disability caused by serious injury is estimated to be 10 times higher for each pediatric injury-related death, and this is an important cause of economic and social burden for patients and families due to missed school time, future work loss, and stigmatization [4,5]. In the United States, unintentional injuries that required medical attention and caused restricted activity affected nearly 20 million children each year between 2000 and 2006. Regarding the economic burden, more than 9 million visits to emergency departments due to pediatric injury resulted in $7.5 billion in medical costs in the United States [6]. The fact that pediatric trauma has a significant impact on patients, families, and countries shows the need for a better understanding and more detailed examination of these cases.

Most deaths due to injury occur during the first minutes after the event, which suggests that prevention strategies are important for decreasing mortality [7]. Accordingly, identifying relevant risk factors is the first step toward prevention. Another way to reduce pediatric injury-related deaths is to take action for improving prehospital care. The main goal is to reduce the duration of transport of injured patients to a hospital in the shortest time with efficient interventions being undertaken [8].

Based on previously published studies, pediatric patients with traumatic and nontraumatic cases transferred by ambulance in developed countries account for 5%–13% of all patients admitted to the emergency department [9,10]. A study performed in four metropolitan cities of Türkiye reported a lower rate of 0.55% for such cases [11]. However, misuse of the emergency medical service (EMS) among all age groups in developed countries such as the United States, Canada, Sweden, and the United Kingdom reaches rates of up to 40%–50% [12–15]. These numbers are twice as high in Türkiye for the pediatric age group [16]. Among trauma patients transported by ambulance, only 17.8% were major trauma patients with an injury severity score (ISS) of >11 [11]. These findings raise the question of how effectively EMS is used.

Statistics on the types of injury, mortality rates, and complications caused by pediatric trauma vary according to the level of development of the country, socioeconomic disparities among regions within the same country, trauma systems, and precautionary actions. Most studies on pediatric trauma have addressed diagnosis, classification, treatment modalities, and patient management, while studies evaluating prehospital pediatric trauma and the use of ambulance services are relatively limited in number [11].

This study aims to determine the demographic characteristics, reasons for admission to the hospital, and diagnoses of pediatric trauma patients who received prehospital emergency health services.

## 2. Materials and methods

### 2.1. Study design

This study was designed as a retrospective observational epidemiological study and included all patients under the age of 18 years who received an ambulance and prehospital emergency healthcare due to trauma and were registered in the Emergency Health Automation System (ASOS) after calls were placed to the 112 emergency call center between 1 January 2018 and 31 December 2022 in Ankara. This study was reviewed and approved by the Yıldırım Beyazıt University Yenimahalle Training and Research Hospital Institutional Review Board (date: 07 December 2023, decision no: E-2023-72). All methods in this study were applied in accordance with the Declaration of Helsinki.

A total of 37,420 patients treated between 2018 and 2022 were included in the study. After an ambulance arrived at the scene, 84.4% of all patients (n = 31,585) were transferred to a hospital by ambulance. While 7.9% of all patients (n = 2957) or their families declined to be transported to the hospital after EMS arrival, 7.6% of the patients were transported to a secondary hospital for specific reasons (n = 2826). The remaining patients were found to be deceased at the scene (n = 17) or experienced cardiac arrest on the way to the hospital and died before or after arrival (n = 35).

After the first evaluation of the patients by the EMS team following the arrival of the team to the scene of injury, patients were transported to either the nearest health center or a specialized center depending on the need for advanced diagnosis and treatment.

Demographic information was collected for all patients, including age and sex. Other variables pertaining to the injury epidemiology, including the reason for calling an ambulance, International Classification of Disease (ICD)-10 diagnosis codes, and injury mechanism, were also collected for all patients. The duration of time to reach the trauma scene, the time spent at the scene, the duration of transport time from the scene to the hospital, reasons for transport between facilities, and the types of districts (urban versus rural) were recorded. Pediatric patients were classified into age groups according to the relevant World Health Organization categorization as <1 year, 1–4 years, 5–9 years, 10–14 years, and 15–18 years.

### 2.2. Statistical analysis

Data analysis was performed using IBM SPSS Statistics 25.0 for Windows (IBM Corp., Armonk, NY, USA). In the evaluation of the data, descriptive variables were presented as percentage, mean, and standard deviation. The compliance of the data with normal distribution was evaluated with the Kolmogorov–Smirnov and Shapiro–Wilk tests. The Pearson chi-square test was used to compare qualitative data, while one-way analysis of variance (ANOVA) and t-tests for independent groups were used to evaluate quantitative data, and Bonferroni and Tukey post hoc analysis was performed for comparisons of multiple groups. Pearson correlation analysis was applied to correlate data that were normally distributed. Statistical significance was accepted at p < 0.05.

## 3. Results

A total of 37,420 patients treated by EMS between 2018 and 2022 were included in this study, and 65.8% (n = 24,627) of these patients were male while 34.2% (n = 12,793) were female. The mean age for all patients was 9.7 ± 5.1 years; for female patients, the mean age was 9.2 ± 5.4 years, and for male patients, it was 10.0 ± 5.0 years. The mean response time (i.e. time interval from the phone call to EMS until arrival at the scene) was 10.4 ± 11.8 min and the mean transport time (i.e. time interval from the ambulance’s departure until arrival at the scene) was 6.2 ± 7.6 min (Table 1). The mean response and transport times were observed to be highest in 2020, while the mean response time was lowest in 2018 and the mean transport time was lowest in 2019.

In comparisons made by year, it was found that there was a statistically significant difference (p < 0.05) between years in terms of the sex and age of the patients, as well as transport times. There was a difference in sex distribution between 2019 and 2022, with the proportion of female patients being higher in 2019. For age, there was a difference in general and for the ages of female patients between 2018 and other years, between 2019 and other years, and between 2019 and 2022. There was a difference in the ages of male patients between 2020 and other years and between 2021 and other years. There was also a difference in transport time (i.e. time from phone call to arrival at the scene) between 2020 and other years, between 2021 and other years, and between 2018 and 2022 (p < 0.001 for all). Finally, there was a statistically significant difference in transport time (i.e. from case assignment to arrival at the scene) between the years of 2018/2019/2022 and 2020/2021, as well as between 2018 and 2020.

While 85.0% (n = 31,809) of all patients who received ambulance service were from urban areas, the remaining 15.0% (n = 5611) of the patients were from rural areas. Compared to other years, the rate of patients transported from rural areas was highest in 2020 at 16.5% (n = 886) and lowest in 2022 at 12.5% (n = 1008). A difference in the rural/urban status of districts was observed between 2022 and all other years (p < 0.001) (Table 1).

After the ambulance arrived at the scene, 84.4% of all patients included in this study (n = 31,585) were transferred by ambulance to a hospital (Table 2). While 47.2% of these patients (n = 14,914) were transferred to a training and research hospital (TRH), 27.0% (n = 8533) were transferred to a state hospital (SH), 24.4% (n = 7720) to a university hospital (UH), and 1.3% (n = 418) to a private hospital (PH). Compared to other hospitals, the transfer rate to TRHs was highest at 49.7% (n = 3794) in 2019 and lowest at 44.2% (n = 3088) in 2022; for SHs, the highest rate was 29.2% (n = 2198) in 2018 and the lowest was 21.9% (n = 1128) in 2021. Over the course of 5 years, the highest rate of transfer to UHs was 28.9% (n = 1488) in 2021 and the lowest was 21.1% (n = 1612) in 2019. When the emergency medical team arrived at the trauma scene, 17 patients were found dead, and 35 patients underwent cardiac arrest after departure from the scene and were proclaimed dead after the administration of cardiopulmonary resuscitation (CPR) efforts. Ambulance transport was declined by 7.9% (n = 2957) of all patients after the first intervention at the scene by the EMS team. In contrast, 7.6% (n = 2826) of all patients transferred by ambulance were transferred between two health facilities. The rate of transport between different facilities was highest in 2020 at 9.2% (n = 492) and lowest in 2022 at 5.8% (n = 467). Most of these interfacility transfers occurred from SHs over the course of 5 years (n = 2223), followed by TRHs (n = 547), PHs (n = 28), and UHs (n = 28), respectively. Most of these transfers took place to a TRH (n = 2.455) or UH (n = 300), followed by PHs (n = 44) and SHs (n = 27) (Table 2).

Between 2018 and 2022, 2336 patients were transferred to other facilities due to the need for specialist care. The reasons for the remainder of the patient transfers to other hospitals were the need for intensive care unit (ICU) admission (n = 251), the need for specific medical equipment (n = 137), and the lack of an empty bed (n = 102). Most interfacility transfers occurred in 2019 (n = 673) and the fewest occurred in 2022 (n = 467) (Table 2).

Between 2018 and 2022, the most frequent reason for admission was falls (n = 26,596). The second most common cause of admission was traffic accidents (n = 4610), followed by sports injuries (n = 1604), injuries involving sharp objects (n = 1045), assault (n = 405), intentional self-injury (n = 105), firearm injuries (n = 46), and other causes (n = 3009) (Table 2).

Pediatric age groups were classified according to the categorization of the World Health Organization as described above in subsection 2.1. During the period of time considered in this study, falls were the most common mechanism of injury in all pediatric age groups (n = 26,596) and traffic accidents ranked second (n = 4610). This was followed by sports injuries (n = 1604), injuries involving sharp objects such as a knife or scissors (n = 1045), assault (n = 405), intentional self-harm (n = 105), and firearm injuries (n = 46). Among all age groups, compared to other types of trauma, falls were the injuries occurring at the highest rate (91.4%, n = 1267) in the group of patients aged <1 year and at the lowest rate (56.6%, n = 4728) in the age group of 15–18 years (Tables 2 and 3). During the course of 5 years, patients were diagnosed with falls more frequently in 2022 (73.4%, n = 5929) and less frequently in 2018 (68.1%, n = 5969). The rate of traffic accidents was highest in 2018 (14.5%, n = 1271) and lowest in 2022 (10.0%, n = 805) (Table 2). Traffic accidents, sports injuries, injuries involving sharp objects, assault, and firearm injuries were most common in the age group of 15–18 years. While an increase was observed in mechanisms of injury other than falls in association with age, a decrease in the number of falls was observed with age (Table 3).

The transport time of the EMS teams from the first contact with the call center to the scene did not vary by age (p < 0.152). Similarly, the time interval from the ambulance’s departure from the center to the scene did not differ statistically between age groups (p < 0.176). However, the difference between age groups in transport duration from arrival at the scene to arrival at the hospital was statistically significant (p < 0.001) (Table 3).

Comparing transport times by sex, it was found that there were no statistically significant differences (p > 0.05) in terms of transport times between sexes in any years (Table 4). The response time from call to arrival at the scene and the duration of transport from the scene to the hospital decreased as the patient’s age increased (r = −0.024, p < 0.001 and r = −0.080, p < 0.001, respectively), while time from the departure of the ambulance to its arrival at the scene increased in positive correlation with age (r = 0.014, p < 0.006) (Table 5).

Among interfacility transfers, TRHs and UHs were the hospitals that received the most patients (n = 2455 and n = 300, respectively). Reviewing the diagnoses of transferred patients, it was found that the most commonly transferred patients were those with fall-related injuries, in direct proportion to the total number of cases (65.0%, n = 1838). Traffic accidents ranked second (16%, n = 453), followed by sports injuries (7.1%, n = 201). The most frequent transfers to PHs involved sports injuries and falls, respectively. The most common diagnoses for patients transferred to TRHs and UHs were fall-related injuries, traffic accidents, and sports injuries, respectively (Table 6).

## 4. Discussion

Injury is a major public health issue in the pediatric age group. It is one of the leading causes of morbidity and mortality, both globally and in Türkiye [1]. The impact of pediatric trauma on patients and their families continues for many years. Since these incidents significantly affect the country’s economy and the quality of life of the society, it is necessary to understand the phenomenon of pediatric trauma thoroughly and examine such cases in detail.

In 1983, Trunkey described injury mortality with a trimodal distribution according to the time interval from injury to death: immediate deaths at the scene, early deaths due to hemorrhage (<4 h from injury), and delayed deaths due to organ failure (>4 h from injury) [17]. Although the pattern of death distribution has changed from trimodal to bimodal in recent years due to improvements in postresuscitation care in the late stage, most deaths due to injury still occur at the scene [7,18]. This suggests that most deaths from pediatric trauma could be prevented by taking relevant precautions. In the present study, when the EMS teams arrived, 17 patients were found dead at the scene of the trauma. Although the short-term mortality of all patients was not analyzed in this study, 35 patients experienced cardiac arrest after the ambulance’s departure from the scene and were proclaimed dead after the administration of CPR (i.e. <4 h from injury).

Identifying risk factors is the first step towards prevention. In a review study, it was reported that preventing trauma in children is very effective and will prevent significant problems impacting patients and their families, healthcare systems, and economies. It was emphasized that such prevention strategies should address all segments of society and be reinforced with compulsory training [19]. The main causes of injuries in children can vary by age; therefore, clarification of the causes of injuries in specific age groups is important. In a study involving Asian countries with different economic statuses, the most common cause of unintentional injuries among those aged 8 and older was traffic accidents, while falls were the predominant cause of injury among children younger than 8 [20]. In a study conducted in Türkiye, the most common causes of trauma in pediatric patients were reported to be traffic accidents and falls, respectively [21]. Similarly, in our study examining cases within specific age groups, we observed that among the cases directed to emergency departments through the 112 ASOS call center, patients aged 10 and over had a higher occurrence of traffic accidents both numerically and percentagewise compared to those younger than 10. Similarly, among patients under 10 years of age, the percentage of falls was higher than among those aged 10 and older. Jung et al. similarly found that the most common causes of trauma among individuals aged 0–4 years were falls and collisions [22]. In the present study, it was observed that the rate of traffic accidents increased with age, while a decrease in the incidence of falls was observed with age. In a study conducted by Oliver et al. to assess the impact of measures taken in the United States, the following changes in the number of cases were revealed: the general rate of hospital admissions linked to child injuries decreased between 2000 and 2011, but there was a growing trend in the proportion of children with severe injuries [23]. Although the rate of ambulance usage for pediatric trauma cases decreased during the COVID-19 pandemic [24], it increased again after the pandemic ended. In the present study, when the ratio of the child population in Ankara in 2018–2022 was examined according to data obtained from TÜİK, no statistically significant differences in terms of incidence rates were found for pediatric trauma cases between the years 2018 and 2022. The lack of an observed change in incidence indicates the need for a greater development of strategies to prevent trauma.

Another way to reduce pediatric injury-related deaths is to take action against deaths in the second period of the trimodal distribution described above, including prehospital care [8]. The paramount goal for decreasing the incidence of death during the second period is transporting injured patients to a hospital in the shortest possible time. This is based on the “golden hour” concept, which holds that delivering trauma patients to definitive care within 60 min optimizes their survival. Accordingly, in Türkiye’s emergency healthcare system, intervention in accordance with the “golden hour” concept is the goal for all trauma patients. Compared to the adult age group, rapid identification and appropriate transport of critically injured patients can be more challenging when dealing with children, especially considering that EMS team members are less frequently in contact with critically ill or injured children [25]. In a study conducted with both pediatric and adult patient groups, it was observed that scene time was shorter for the pediatric and male patient groups [26]. However, in our study, no significant differences were observed between male and female groups of patients for transport times (from time of the call to the ambulance’s arrival at the scene, from ambulance departure to scene, and from scene departure to hospital arrival) in any given year (Table 4). Regarding the comparison of transport times among age groups, except for a positive relationship between the duration from scene arrival to hospital arrival and age (p < 0.001), no significant results were obtained. Although previous research indicating a similar difference was not designed to analyze the reasons, some possibilities were noted: first, as the patient’s age decreases, EMS team members who have not encountered significant numbers of patients in that age group may hesitate to perform field interventions, especially in critical pediatric cases, and may prefer to transport such patients to the hospital immediately, and second, it is easier to extract patients with smaller body sizes from difficult conditions and carry them for transport [27]. Sampalis et al. found that hospitalization after the golden hour was associated with a 3-fold increase in mortality, and for each additional minute of prehospital time, the risk of mortality increased by 5% [28]. Similarly, a study conducted by Petri et al. concluded that more out-of-hospital time was associated with decreased survival [29]. However, more recent studies have shown groundbreaking results on this subject. Lerner and Moscati analyzed approximately 2000 trauma records and concluded that age and injury severity were associated with mortality. However, the duration of time before hospitalization was not associated with mortality [30]. Similarly, Newgard et al. conducted a multicenter study of prehospital time and found no correlation between prehospital time and survival in adult patients [31]. In this study, the survival rate could not be examined due to the lack of access to relevant hospital records. Although it is well studied in the adult population, the role of the golden hour in pediatric trauma remains unclear.

The accurate application of field evaluation protocols by prehospital care providers is crucial for quickly identifying severely injured patients in the field and transporting them accordingly. However, even after the initial transport, some pediatric patients may still require secondary transport to specialty hospitals for definitive care. Although transferring patients to a more appropriate facility may be beneficial for the patient’s prognosis, secondary transport often worsens physiological instability due to transport stresses or progression of the disease. Children are particularly vulnerable to adverse events related to transport [32]. Some studies have shown that patient outcomes at pediatric trauma centers (PTCs) reflect decreased morbidity and mortality [33]. However, requiring every pediatric trauma patient to go to a PTC may impose an unnecessary burden on those centers. Therefore, the primary objective should be to properly triage patients and transport those who require specialty care to the appropriate centers. Most patients undergoing secondary transport are individuals of younger age groups with conditions such as burns, nonaccidental trauma, head/neck injury, and multiple injuries [25]. In this study, the most common reasons for these transfers were falls, traffic accidents, and sports injuries. Transfers predominantly occurred to tertiary hospitals (TRHs and UHs), and 6.2% (n = 2336) of the 37,420 analyzed patients were transferred to another hospital due to the need for specialist care as a result of the lack of specialties such as neurosurgery, orthopedics, otorhinolaryngology, and pediatric surgery in the centers in which the patients were first evaluated. However, Öztan et al. argued that secondary overtriage in Türkiye emerges from legal obligations to perform consultations and it does not influence patient outcomes [26]. Insufficient prehospital and in-hospital guidelines for triaging severely injured children result in missed injuries, necessitating secondary transfers and the inefficient use of limited resources [34]. Improvements in pediatric trauma triage are needed to effectively pinpoint patients who would benefit the most from direct transport to a PTC. However, the creation of a perfect tool for this purpose is quite challenging. Therefore, prehospital pediatric classification guidelines should be continually evaluated and refined [35].

This study has some limitations. Since the data for this study were obtained from the 112 ASOS system, not from hospital records, the final mortality rates and the diagnoses of the patients after hospital admission could not be analyzed. Similarly, the 112 ASOS data did not provide information about the diagnosis with which trauma patients were transported to advanced centers. Data from the autopsies of patients who were proclaimed dead were not available. Although physiological data for patients at the time of the ambulance’s arrival were available to us, they were not analyzed in the study because they did not include sufficient parameters for objective scoring such as the ISS.

Targeted preventive measures and community education should address the specific causes of trauma that are more prevalent in certain age groups [7]. Early identification of special patient groups that require secondary transport can reduce mortality and morbidity related to trauma through direct transfer of these patients to appropriate hospitals [34]. As was recommended in a review study of prehospital trauma triage systems [36], region-specific specialized groups for pediatric trauma transport should be established and receive regular training on updated guidelines for knowledge improvement. Protocols to prevent primary and secondary overtriage should be established based on research findings.

## Figures and Tables

**Table 1:** Demographics, response times, and transport times by years.

Variables		Total (n = 37,420)	2018 (n = 8763)	2019 (n = 8960)	2020 (n = 5355)	2021 (n = 6262)	2022 (n = 8080)	p
**Sex**	Male	24,627 (65.8%)	5781 (66.0%)	5791 (64.6%)	3504 (65.4%)	4132 (66.0%)	5419 (67.1%)	**0.020** a
	Female	12,793 (34.2%)	2982 (34.0%)	3169 (35.4%)	1851 (34.6%)	2130 (34.0%)	2661 (32.9%)	
**Age (years)**		9.7 ± 5.1	10.0 ± 5.2	9.9 ± 5.0	8.7 ± 5.4	9.3 ± 5.2	10.2 ± 4.8	**<0.001** b
	Female	9.2 ± 5.4	9.6 ± 5.4	9.3 ± 5.2	7.9 ± 5.6	8.7 ± 5.3	9.8 ± 5.1	**<0.001** b
	Male	10.0 ± 5.0	10.2 ± 5.0	10.1 ± 4.9	9.2 ± 5.3	9.6 ± 5.2	10.3 ± 4.7	**<0.001** b
**Response time** (minutes from call to scene)		10.4 ± 11.8	9.7 ± 11.6	10.1 ± 13.6	11.7 ± 12.5	11.0 ± 9.2	10.0 ± 11.0	**<0.001** b
**Transport time (minutes from ambulance departure to scene)**		6.2 ± 7.6	6.2 ± 7.2	6.1 ± 9.0	6.5 ± 8.2	6.5 ± 5.4	6.1 ± 7.1	**<0.001** b
**District**	Urban	31,809 (85.0%)	7386 (84.3%)	7571 (84.5%)	4469 (83.5%)	5311 (84.8%)	7072 (87.5%)	**<0.001** a
	Rural	5611 (15.0%)	1377 (15.7%)	1389 (15.5%)	886 (16.5%)	951 (15.2%)	1008 (12.5%)	

a chi-square test (n, %);

b one-way ANOVA (mean ± SD).

**Table 2:** Diagnoses and features of transfers by years.

Features of transfers		Total (n = 37,420)	2018 (n = 8763)	2019 (n = 8960)	2020 (n = 5355)	2021 (n = 6262)	2022 (n = 8080)
**Transport to hospital**		31,585 (84.4%)	7521 (85.8%)	7631 (85.2%)	4307 (80.4%)	5147 (82.2%)	6979 (86.4%)
	TRH	14,914 (47.2%)	3519 (46.8%)	3794 (49.7%)	2036 (47.3%)	2477 (48.1%)	3088 (44.2%)
	SH	8533 (27.0%)	2198 (29.2%)	2150 (28.2%)	1094 (25.4%)	1128 (21.9%)	1967 (28.2%)
	UH	7720 (24.4%)	1629 (21.7%)	1612 (21.1%)	1145 (26.6%)	1488 (28.9%)	1841 (26.4%)
	PH	418 (1.3%)	175 (2.3%)	75 (1.0%)	32 (0.7%)	54 (1.0%)	83 (1.2%)
**Transport declined**		2957 (7.9%)	596 (6.8%)	644 (7.2%)	547 (10.2%)	546 (8.7%)	624 (7.7%)
**Interfacility transfer**		2826 (7.6%)	633 (7.2%)	673 (7.5%)	492 (9.2%)	561 (9.0%)	467 (5.8%)
From:	SH	2223 (78.7%)	480 (75.8%)	529 (78.6%)	381 (77.4%)	445 (79.3%)	388 (83.1%)
	TRH	547 (19.4%)	136 (21.5%)	135 (20.1%)	103 (20.9%)	106 (18.9%)	67 (14.3%)
	PH	28 (1.0%)	11 (1.7%)	2 (0.3%)	3 (0.6%)	4 (0.7%)	8 (1.7%)
	UH	28 (1.0%)	6 (0.9%)	7 (1.0%)	5 (1.0%)	6 (1.1%)	4 (0.9%)
To:	TRH	2455 (86.9%)	551 (87.0%)	589 (87.5%)	426 (86.6%)	457 (81.5%)	432 (92.5%)
	UH	300 (10.6%)	61 (9.6%)	73 (10.8%)	57 (11.6%)	81 (14.4%)	28 (6.0%)
	PH	44 (1.6%)	5 (0.8%)	5 (0.7%)	8 (1.6%)	20 (3.6%)	6 (1.3%)
	SH	27 (1.0%)	16 (2.5%)	6 (0.9%)	1 (0.2%)	3 (0.5%)	1 (0.2%)
**Ex**	Arrest at scene	17 (0.0%)	3 (0.0%)	3 (0.0%)	4 (0.1%)	5 (0.1%)	2 (0.0%)
	Arrest before reaching hospital	35 (0.1%)	10 (0.1%)	9 (0.1%)	5 (0.1%)	3 (0.0%)	8 (0.1%)
**Reasons for interfacility transfer**							
Need for specialist		2336 (82.7%)	525 (82.9%)	552 (82.0%)	418 (85.0%)	460 (82.0%)	381 (81.6%)
Need for ICU		251 (8.9%)	67 (10.6%)	66 (9.8%)	38 (7.7%)	40 (7.1%)	40 (8.6%)
Need for medical equipment		137 (4.8%)	24 (3.8%)	31 (4.6%)	18 (3.7%)	33 (5.9%)	31 (6.6%)
Lack of empty bed		102 (3.6%)	17 (2.7%)	24 (3.6%)	18 (3.7%)	28 (5.0%)	15 (3.2%)
**Diagnosis/trauma mechanism**							
Fall		26,596 (71.1%)	5969 (68.1%)	6405 (71.5%)	3802 (71.0%)	4491 (71.7%)	5929 (73.4%)
Traffic accidents		4610 (12.3%)	1271 (14.5%)	1112 (12.4%)	639 (11.9%)	783 (12.5%)	805 (10.0%)
Sports injury		1604 (4.3%)	359 (4.1%)	342 (3.8%)	235 (4.4%)	276 (4.4%)	392 (4.9%)
Injury with sharp object		1045 (2.8%)	258 (2.9%)	210 (2.3%)	183 (3.4%)	170 (2.7%)	224 (2.8%)
Assault		405 (1.1%)	112 (1.3%)	96 (1.1%)	46 (0.9%)	80 (1.3%)	71 (0.9%)
Intentional self-harm		105 (0.3%)	30 (0.3%)	20 (0.2%)	8 (0.1%)	11 (0.2%)	36 (0.4%)
Firearm injury		46 (0.1%)	13 (0.1%)	10 (0.1%)	14 (0.3%)	3 (0.0%)	6 (0.1%)
Others		3009 (8.0%)	751 (8.6%)	765 (8.5%)	428 (8.0%)	448 (7.2%)	617 (7.6%)

TRH: training and research hospital; SH: state hospital; PH: private hospital; UH: university hospital; Ex: exitus.

**Table 3:** Duration of transport and diagnoses by age groups.

Transport duration (min)	Total (n = 37,420)	<1 year (n = 1386)	1–4 years (n = 6293)	5–9 years (n = 8788)	10–14 years (n = 12,604)	15–18 years (n = 8349)	p
From call to arrival at the scene*	9.6 ± 8.5	9.7 ± 16.5	9.3 ± 8.2	9.6 ± 7.2	9.7 ± 9.0	9.6 ± 7.2	0.152^a^
Ambulance departure to arrival at scene*	6.2 ± 5.9	6.2 ± 6.4	6.1 ± 5.3	6.3 ± 5.2	6.3 ± 6.8	6.2 ± 5.2	0.176^a^
Arrival at scene to hospital arrival*	11.7 ± 9.2	12.1 ± 8.3	12.3 ± 8.7	12.1 ± 8.3	11.5 ± 8.8	11.2 ± 10.9	**<0.001** a
**Diagnosis/trauma mechanism**							
Fall	26,596 (71.1%)	1267 (91.4%)	5224 (83.0%)	6575 (74.8%)	8818 (70.0%)	4728 (56.6%)	**<0.001** b
Traffic accident	4610 (12.3%)	63 (4.5%)	395 (6.3%)	1128 (12.8%)	1582 (12.6%)	1444 (17.3%)	
Sports injury	1604 (4.3%)	0 (0.0%)	197 (3.1%)	322 (3.7%)	643 (5.1%)	424 (5.1%)	
Injury with sharp object	1045 (2.8%)	6 (0.4%)	72 (1.1%)	173 (2.0%)	346 (2.7%)	448 (5.4%)	
Assault	405 (1.1%)	5 (0.4%)	5 (0.1%)	14 (0.2%)	141 (1.1%)	240 (2.9%)	
Intentional self-harm	105 (0.3%)	0 (0.0%)	2 (0.0%)	2 (0.0%)	36 (0.3%)	64 (0.8%)	
Firearm injury	46 (0.1%)	0 (0.0%)	2 (0.0%)	5 (0.1%)	12 (0.1%)	27 (0.3%)	
Others	3009 (8.0%)	45 (3.2%)	396 (6.3%)	569 (6.5%)	1026 (8.1%)	974 (11.7%)	

a one-way ANOVA (mean ± SD);

b chi-square test (n, %);

* for patients transported from the scene.

**Table 4:** Comparison of transport durations by sex.

Years	Transport duration (minutes from departure from scene to hospital)		p*
	Female	Male	
2018 (F = 2982, M = 5781)	13.0 ± 12.8	12.9 ± 14.4	0.701
2019 (F = 3169, M = 5791)	13.3 ± 16.4	12.8 ± 16.9	0.184
2020 (F = 1851, M = 3504)	15.5 ± 14.9	15.1 ± 13.7	0.313
2021 (F = 2130, M = 4132)	16.7 ± 17.2	16.0 ± 15.5	0.116
2022 (F = 2661, M = 5419)	13.3 ± 11.6	13.5 ± 14.0	0.701
Total (F = 12,793, M = 24,627)	14.6 ± 0.1	15.1 ± 0.1	0.086

* independent samples t-test; F: female; M: male.

**Table 5:** Correlations of age with transport duration.

Transport duration	Age (years)	
	r	p*
Response time (minutes from call to arrival at scene)	−0.024	**0.000**
On-scene time (minutes from ambulance departure to scene)	0.014	**0.006**
Transport duration (minutes from scene departure to hospital arrival)	−0.080	**0.000**

* Pearson’s correlation test.

**Table 6:** Diagnoses and hospitals for interfacility transfers.

Diagnosis/trauma mechanism	Total (n = 2826)	SH (n = 27)	TRH (n = 2455)	PH (n = 44)	UH (n = 300)
Fall	1838 (65.0%)	13 (48.1%)	1626 (66.2%)	17 (38.6%)	182 (60.7%)
Traffic accident	453 (16.0%)	4 (14.8%)	378 (15.4%)	21 (47.7%)	50 (16.7%)
Sports injury	201 (7.1%)	1 (3.7%)	181 (7.4%)	3 (6.8%)	16 (5.3%)
Injury with sharp object	48 (1.7%)	2 (7.4%)	39 (1.6%)	0 (0.0%)	7 (2.3%)
Firearm injury	6 (0.2%)	0 (0.0%)	6 (0.2%)	0 (0.0%)	0 (0.0%)
Intentional self-harm	6 (0.2%)	0 (0.0%)	4 (0.2%)	0 (0.0%)	2 (0.7%)
Assault	3 (0.1%)	0 (0.0%)	2 (0.1%)	0 (0.0%)	1 (0.3%)
Others	271 (9.6%)	7 (25.9%)	219 (8.9%)	3 (6.8%)	42 (14.0%)

TRH: training and research hospital; SH: state hospital; PH: private hospital; UH: university hospital.

## Footnotes

1 TÜİK (2023). İstatistiklerle Çocuk, 2022 [online; in Turkish]. Website: https://data.tuik.gov.tr/Bulten/Index?p=Istatistiklerle-Cocuk-2022-49674 [accessed 15 April 2024].
